# Predictive Validity of Interviewer Post-interview Notes on Candidates’ Job Outcomes: Evidence Using Text Data From a Leading Chinese IT Company

**DOI:** 10.3389/fpsyg.2020.522830

**Published:** 2021-01-07

**Authors:** Shanshi Liu, Yuanzheng Chang, Jianwu Jiang, Haigang Ma, Huaikang Zhou

**Affiliations:** ^1^School of Business Administration, South China University of Technology, Guangzhou, China; ^2^Business School, Guilin University of Technology, Guilin, China; ^3^College of Management, Shenzhen University, Shenzhen, China; ^4^Tencent Holdings Limited, Shenzhen, China

**Keywords:** selection interview, structured interview, text mining, narrative comments, job analysis, career performance

## Abstract

Despite the popularity of the employment interview in the employee selection literature and organizational talent selection process, few have examined the comments interviewers give after each interview. This study investigated the predictability of the match between interviewer post-interview notes and radar charts from job analysis on the candidate’s later career performance using text mining techniques and data from one of the largest internet-based technology companies in China. A large sample of 7,650 interview candidates who passed the interviews and joined the company was obtained to show that the number of job-related capabilities interviewers mentioned in their notes was positively related to candidate’s job performance, the number of promotions, and negatively related to turnover. Moreover, the dimensions of the radar chart from job analysis covered in the interview moderated the predictability of interview post-interview notes. Our results indicated that a smaller number of radar chart dimensions by which interviewers assessed the candidates in the interview positively moderated candidates’ promotion for product development jobs, and negatively moderated turnover for technical jobs. The implications of these results for structured interview research in both theory and practice are discussed.

## Introduction

The employment interview is a proven and popular selection method that has drawn continuous attention from researchers for more than 100 years (Macan, 2009). It is often used to assess the fit of the candidate to the employer and is shown to have high predictive validity for job performance (Huffcutt et al., 2001). One of the most consistent findings of selection interviews is that structured interviews are more reliable and valid than unstructured interviews (Levashina et al., 2014). Extant literature focuses on the factors that influence interviewers’ adoption of interview structure and the structured interview process itself, trying to give better guidance in terms of how to increase its adoption and the boundary of when to use it. For example, Chapman and Zweig (2005) find that interviewer training, interview focus, interviewer reactions to interview structure, and applicant reaction to interview structure lead to different levels of interview structure and how interviewees react to them. Campion et al. (1997) identified 15 components of the interview structure and divided them into two categories: what to ask and how to evaluate. The reliability, validity, and user reactions of these components were assessed. Huffcutt and Arthur (1994) proposed a framework that defined interview structure with similar categories: standardized interview questions, and standardized response evaluation. Their framework divided interview structure into four levels, with level 1 being structure with no formal constraints to level 4 being asking the same questions with no modifications allowed. Particularly, level 2 was defined as “limited constraints, typically standardization of the topical areas to be covered.” However, interview structure is still often utilized dichotomously in many studies, mostly comparing high interview structure with no structure. Despite this framework, few studies tried to explore the level 2 interview structure on the outcome of a candidate’s job performance.

Among the many components of interview structure, job analysis is utilized most frequently in interview structure research for interview questions content dimension (Levashina et al., 2014). Job analysis serves as a foundation of selection in human resources and one of the two main goals of job analysis is to find out what kind of worker fits best to certain jobs (Sanchez and Levine, 2012). For the job analysis component, finding accurate job requirements is the key to develop relevant interview questions to assess a candidate’s capabilities. Collecting critical job incidents with job experts, such as managers and interviewers, is the most common method (Morgeson et al., 2016). However, the development of job-related structured interview questions in previous research is often carried out by researchers, not professionals in the field (Campion et al., 1997), therefore not reflecting the full picture of this process in real situations.

On the other hand, interview structure can be grouped by how note-taking is required in the interview process, such as whether notes are taken during or after the interview or what to write in the notes. Campion et al. (1997) suggest that taking notes during the interview is considered a higher structure level than after the interview. On the other hand, taking notes in great detail during the interview is considered the highest level, while note-taking without specific instructions about when and how to write notes being the less constrained structure. Although studies have shown that taking notes during or after an interview helps interviewers organize their thoughts, thus making better judgments about the candidate, few have tried to explore the content of these notes and how it relates to the candidate’s job performance.

Apart from the interview structure, the Person-Job (PJ) fit theory suggests that finding the match between the job candidate’s attributes and the job requirements is the cornerstone of a successful selection (Barrick and Parks-Leduc, 2019). Hu et al. (2016) show that supervisors’ Person-Organization fit perceptions are positively related to new hires’ performance for executives in a Fortune 500 company. How the fit between the candidate and the job predicts a candidate’s future job performance remains an unanswered question (Posthuma et al., 2002). Interview validity is shown to increase if questions asked during a structured interview are job-related (Wiesner and Cronshaw, 1988) because it can better assess if the candidate fits the job requirements. However, only indirect effects have been found between job analysis and interview validity (Conway et al., 1995). Extant research mostly uses cognitive test results as the dependent variable to show the validity of using job analysis in structured interviews (Thorsteinson, 2018). These cognitive performance scores are then linked to the candidate’s job performance. However, whether using job analysis as an interview component directly affects interview validity requires further exploration.

This paper aims to investigate the direct effect of interviewer notes on interview validity, while focusing on the role of job analysis as an interview structure component. The notes we use in our study are the narrative comments interviewers write after each interview about what they think of the candidate for later reference as required by the company. What interviewers say about the candidate reflects the questions interviewers asked or cared the most about during the interview because the comments consist of the evaluation of the answers candidate provided during the interview. This is shown by the appearance of job-related keywords in their notes. For example, if an interviewer wants to know whether the candidate has good leadership skills, he/she may ask the candidate through direct or indirect questions, such as “how do you lead a team” or “tell me an event you organized.” The interviewer would write down how he/she thought about the candidate’s performance in the note with the keyword leadership skills. Examining these keywords allows us to get insights about what the interviewer what to know during that interview. Specifically, we examine: (1) whether the match between the interviewer’s post-interview note about the candidate’s interview performance and the job requirements of the position will predict the candidate’s job performance, promotion, and turnover and (2) how interviewers could utilize the radar chart from job-analysis to better assess interviewees for different types of jobs.

Our study extends the interview literature in three ways. First, we draw from interviewers’ narrative comments in their post-interview notes in actual scenarios to reflect the questions interviewers go through during the interview, which shows the direct effect of interview notes on interview validity. Moreover, while PJ fit is shown to predict higher job satisfaction, better job performance, and lower turnover rate (Kristof-Brown et al., 2005), our study also includes candidates’ number of promotions, making it a well-rounded measurement of fit and interview validity.

Second, the practices the company used in our study required interviewers to assess the candidate using their way of question-asking and evaluation without following a strict structure. The questions interviewers asked during an interview were either self-reported or pre-determined in previous studies, which might not reveal the situation in an actual interview. Our study puts less emphasis on the structure of interviews in favor of what interviewers know about the candidate’s job-related capabilities by examing the content of interviewer narrative comments in post-interview notes with the radar chart from job analysis rather than what structure interviewers follow during the interview. This is similar to the level 2 of interview structure proposed by Huffcutt and Arthur (1994) such that radar charts serve only as a framework for interview questions without providing exact questions to be asked. With the results of this study, interviewers and practitioners could use the scores and attributes of the job analysis result in interviews as a reference for their questions without sticking to pre-determined questions to evaluate candidates. This approach extends structured interview research by showing how level 2 of both dimensions proposed by Huffcutt and Arthur (1994, p. 186), “characterized by limited constraints, typical standardization of the topical areas to be covered,” affects interview validity.

Third, despite the emergence of the big data movement in other management contexts (Kobayashi et al., 2018), studies on employment interviews have mostly maintained the use of traditional datasets and methods. Besides, many studies on interview validity use mock interviews or experiments to test their hypotheses, which have been shown to have lower validity than real job interviews (Posthuma et al., 2002). Our study uses text analysis and natural language processing methods with data generated in real practices to explore the content of what interviewers say after the interview, which extends the use of text mining in explanatory research/hypotheses testing in employment interview literature (Kobayashi et al., 2018).

## Conceptual Background and Hypotheses Development

### The Match Between Interviewers’ Narrative Comments and Radar Charts Dimensions on the Interview’s Predictive Validity

Whether interviews can predict a candidate’s job performance has long been of interest to researchers and professionals. Huffcutt et al. (2001) find from multiple meta-analyses that interview is a valid selection method for candidates’ job performance prediction. One of the main research areas in selection interviews is the effect of interview structure on interview validity. Job analysis is one of the components used most frequently in structured interviews (Levashina et al., 2014). One of the essential roles of job analysis is to figure out the work attributes required to perform well on the job (Sanchez and Levine, 2001). A job analysis reveals the requirements of the job and therefore helps identify a matched candidate that fits the role complementarily (Muchinsky and Monahan, 1987). It serves as the foundation on which interviewers and professionals can base their interview questions, which is shown to increase interview validity (Wiesner and Cronshaw, 1988). Radar charts with job requirements and their relative weights were developed by experts from the company as references for employee selection and improvement.

**Figure 1:**
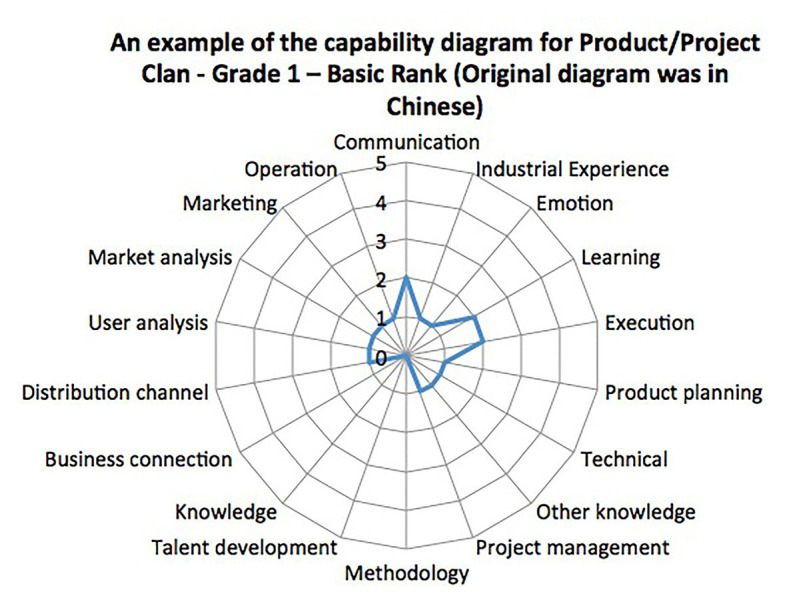
Job-analysis diagram example downloaded from company website.

In our study, job analysis is done by company human resource business partners who understand both the business objectives and human resource practices. They first discuss the goal of each position in the business unit with the unit leader to determine the capabilities needed and a weight is assigned to each of these capabilities. Greater weight means a higher likelihood to contribute to a better performance grade. A radar chart of each of the positions is then generated to make it easier for others to understand and utilize (Figure 1). More specifically, the radar chart here in our study is a chart with capabilities needed for the position and their weights arranged around a circle with lines plotted from the center to the edge. The longer the line the higher the weight of the capability with the name indicated at the edge. One of the advantages of radar charts is that they allow users to evaluate the capabilities and weights simultaneously, which is supposed to help interviewers form their interview questions. By using the radar chart together with proper interview training on how to ask questions, interviewers are more likely to focus on the capabilities needed for the position and ask the pivotal questions.

Another important component of the interview structure is notes written by interviewers. Interviewer notes contain rich text data about what they think of the candidate’s performance during the interview. Text data, a new kind of qualitative data, has become available to researchers in human resources (e.g., Platanou et al., 2018), thanks to the development of information technology. In particular, narrative comments from supervisors have shown their validity in performance appraisal literature as a new data source. Comparing to traditional performance ratings, comments in notes offer more contextual information and require a higher cognitive process to write so that are less vulnerable to social and cultural rating impacts (Brutus, 2010). Moreover, some articles have shown the validity of using text data in personnel selection study, such as candidate essays (e.g., Campion et al., 2016) and admission interviews (e.g., Elam et al., 1994). Given the proximity between performance appraisal and selection interview in human resource management research under the broad category of assessing people (Markoulli et al., 2017), interviewers’ narrative comments in their notes after the interview about a candidate’s performance provide a different and unique perspective on selection interview research.

One of the purposes of selection interviews is to assess the fit, where PJ fit refers to the compatibility between the candidate’s characteristics and the requirements of the job (Kristof-Brown et al., 2005). The framework of PJ fit proposed by Edwards (1991) consists of two classes: needs and supplies (NS) fit, and demands and abilities (DA) fit. NS fit focuses on the desire of the employee and what the job can provide. DA fit focuses on job demands and what the employee’s capabilities have to offer. Interviewers assess candidates’ job-related capabilities by asking job analysis related questions, such as using capabilities chart or opinion from experts as references. Although the narrative comments in the notes may not reveal the actual opinion of the interviewer on the aspect of the job requirements radar chart, it is reasonable to assume that the candidate has met the criteria since our data only have the candidates who passed the interview. Because of the different conceptualization, NS fit is often utilized in studies to examine its relationship with job satisfaction, job stress, and motivation, while DA fit is utilized as a predictor of job performance, promotion, and retention (Edwards, 1991). Boon and Biron (2016) find that higher DA fit predicts higher turnover intention when leader-member exchange (LMX) quality is high. Kristof-Brown et al. (2005) show in their meta-analysis that PJ fit is a valid predictor for job performance and turnover, where the correlation coefficient is positive and negative, respectively. There are three common measurement methods for PJ fit: perceived fit, subjective fit, and objective fit. Interviews serve as a crucial tool to assess the fit of a candidate before they are selected into the organization. Interviewers can assess the match between a candidate’s values and the organization with high accuracy (Cable and Judge, 1997).

Interview narrative comments in our study are the content interviewers write down in their notes after an interview to record what they think of the candidate. They serve as a tool for interviewers to give a review of the candidate’s response to the questions the interviewer asked during the interview. Since interviewer narrative comments are a kind of performance appraisal that assesses people’s behaviors, they can be scored into different performance dimensions (Speer et al., 2018). Interviewers in our research are required to write notes after each interview by their human resources system. The notes are without format or content restriction. Interviewers write down their impression of the candidate’s interview performance and answers to their questions to support their decision on whether the candidate pass or fail. They are expected to write in more detail. By investigating the dimensions covered in the comments, we will be able to know what qualities of the candidate the interviewer has examined during the interview. If the interviewer asked job-related questions during the interview and was able to assess the fit between the candidate and job requirements, the interview will better predict the candidate’s job performance, promotion, and turnover. Therefore, we proposed the following hypotheses:

*H1*: The match between the job requirements radar chart aspects and the interviewer’s narrative comments on the candidate positively relates to the candidate’s (a) job performance, (b) the number of promotions, and negatively relates to the candidate’s (c) turnover.

### The Effect of the Breadth of Interview Questions on the Matching Score Between Interviewers’ Narrative Comments and Radar Chart Dimensions on the Interview’s Predictive Validity

Although using the radar chart from job analysis to construct interview questions increases interview validity, how to use the chart more effectively needs further investigation. Since longer interviews cost the organization more (Thorsteinson, 2018), interviewers are not likely to spend much time on an interview. Within a limited length of interview time, interviewers can either go broader to cover more aspects of the radar chart from job analysis and get a comprehensive impression of the candidate or select certain aspects to get a deeper understanding of the candidate’s capabilities. Due to the trade-offs between time and effort, it would be hard for individuals to have broad knowledge domains and be specialized in all of them at the same time. The breadth and depth of employee knowledge are utilized as the two ends of one dimension (Schilling et al., 2003). Since the job requirements across positions vary, and the depth and breadth of knowledge affect how employees perform on the job, the choice of interviewers’ questioning strategy may have different effects on interview validity.

To compare the breadth effect across different job groups, we used all the entry-level jobs of an internet-based technology company that were different in roles and job requirements. The company has divided its jobs into five categories, called Clans: Technical, Product/Project, Marketing, Specialist, and Design. As these names suggest, jobs are grouped into different Clans based on their skill requirements. For example, jobs in the Technical Clan are mainly engineers that require programming and computer skills, whereas jobs in the Marketing Clan are jobs that require business skills for the company’s sales of products and business expansion. Boh et al. (2014) found in their research that the breadth of employee expertise leads to a higher number of inventions, while the depth of expertise is more helpful for inventions that are more complicated and technical. Employees with deeper knowledge would be able to recombine their existing knowledge to have a significant impact on technological change (Fleming, 2001).

Technical jobs often involve abstract problem solving and logical reasoning, therefore a deeper understanding of the tools and logic of completing the tasks will allow employees to perform better. Since our data were from an internet-based technology company whose products are mainly social media platforms and games for consumers where competition is intense, the company needs the products they develop to stand out and differentiate themselves from other competitors. This product development process requires product managers to have a comprehensive and deep understanding of the market and user base, where the depth of knowledge is crucial. Positions in the Specialists Clan include many functions such as legal, investor relations, and enterprise management. These jobs are highly specialized and often require employees to participate in training courses or to obtain licenses before being allowed to perform. For jobs in these Clans, interviewers should focus more on the specialized capability requirements and narrow down their attribute dimensions on the radar charts to get a more detailed understanding of the candidate’s proficiencies to complete specific tasks. Therefore, we proposed the following hypotheses:

*H2*: The relationship between the appearance of radar chart related keywords in the interviewer’s narrative comments and the candidate’s job performance is moderated by the number of dimensions the interview covered, such that the fewer the dimensions, the (a) better performance score, (b) higher number of promotions, and (c) lower turnover probability of the candidate for the Technical, Product/Project, and Specialists Clans.

On the other hand, the breadth of employees’ knowledge enables them to restructure their expertise in innovative ways (Boh et al., 2014). Simonton (2003) finds in his study that the higher number and variety of different cognitive elements available for association increase the probability of generating creative combinations. His result indicates that breadth of knowledge helps individuals to have new ideas and be more creative. This is especially important for employees in the Design Clan, whose jobs are drawing and graphic design for games and social media applications. The design process usually involves recombining different elements and features from different domains to create something new. Thus, employees’ breadth of knowledge could help expand their search domains and come out with different features and styles to fit the themes of different applications.

Jobs in the Marketing Clan are mostly roles in the business operation of the company, such as marketing, sales, and strategic planning. These jobs do not require very technical or specialized skills but do call for the ability to come out with new ideas for the changing business environment. Besides, business operations often involve different parties and their interests. Solving business problems requires building connections with these parties to communicate effectively. Since these parties are different and their interests vary, the breadth of knowledge will help employees to increase the variety of ideas to handle different scenarios and novel business problems (Greve and Taylor, 2000). Because the nature of the jobs in the Design and Marketing Clans is different and requires more innovation but less technical know-how, the breadth of knowledge is more critical here than the other three Clans. Therefore, we proposed the following hypotheses:

*H3*: The relationship between the appearance of radar chart related capabilities keywords in the interviewer’s narrative comments and the candidate’s job performance is moderated by the number of dimensions the interview covered, such that the more the dimensions, the (a) better performance score, (b) higher number of promotions, and (c) lower turnover probability of the candidate for the Design and Marketing Clans.

## Materials and Methods

### Data and Procedure

We tested our hypotheses using human resources data from one of the top internet-based technology companies in China. This company has a well-built data infrastructure that supports data from many human resource departments and functions, including recruitment, assessment, training, compensation, and turnover management. We were able to link these different systems using the unique id number of each candidate that has been selected into the company after the interviews.

All the jobs in the company are divided into five Clans mentioned above. The hierarchy is Clan – Class – Position. For example, a typical hierarchy could be Technical Clan – Technology Research and Development Class – Web Front-End Development Position. Also, each position can be further classified into different Grades and Ranks. There are 6 Grades in total, which follow the hierarchy as Entry – Intermediate – Specialist – Expert – Master – Fellow, and are labeled 1–6. Furthermore, each Grade has three Ranks: Basic, Regular, and Professional. For any employee to be promoted to the next Grade, he/she has to go through the Ranks one by one, from Basic to Professional. For each Rank, there is a dedicated capability radar chart from job analysis that is used as a training reference for both the supervisor and employee to understand the requirements of the position at that level. An example of the diagram is given below (Figure 1).

All candidates seeking a job in this company have to be interviewed. This company has an interviewer scheme program where employees have to go through a training class before being allowed to interview job applicants. The training class aims to familiarize employees with the interview process and remind them to not ask interview questions that may impose legal issues such as questions related to gender, marital status, and age. In the training materials, we obtained from the company’s internal knowledge-sharing platform, it specified that interviewers should adopt the Situation, Task, Action, Result (STAR) interview method to assess interviewees’ capabilities and experiences. This method requires interviewers to be less specific and ask more open-ended questions so that they can listen to and observe more carefully the answers given by the interviewee. After each interview, interviewers are required to write down their thoughts about the candidate. There is no pre-specified structure of the comments; interviewers can determine what to write and in what detail, although giving clear and concise comments is encouraged. This requirement together with the STAR interview method makes the interview comments reasonable evidence of what capabilities and experiences the interviewer assessed the candidate during the interview and in what detail.

The sampling frame for this study consisted of 7,650 candidates who had gone through the selection process and had become a formal employee of the company from 01 July 2016 to 01 July 2018. These candidates applied for various positions, including 3660 (47.8%) candidates for Technical Clan, 1825 (23.9%) candidates for Product/Project Clan, 1268 (16.6%) candidates for Marketing Clan, 449 (5.9%) candidates for Specialist Clan, and 447 (5.9%) candidates for Design Clan. The average age of the candidates is 31.16, with 75.4% male. The distribution of the job applications for each Clan reflects the actual employee composition of the company, as programmers and product managers are the most needed talents in an internet-based company whose products are mainly social media applications and online games.

### Measures

#### Performance Rating

Employees receive a job performance rating from their immediate supervisor biannually at the team level. This rating is called “star” and is given out on a five-point scale, ranging from 1 star being unacceptable to 5 stars being exceptional. We used the first term rating as the measure of employee performance rating because it was the first rating the candidate received after the interview, which reflected the interview assessment of the fit between the candidate and the dimensions from the radar chart more accurately, as the predictability decreases over time (Barrick and Zimmerman, 2009).

#### Promotion

A promotion is defined as a move to a higher Rank in the hierarchy described above. The number of promotions is recorded by counting the upward change in employee position level during the time he or she is in the company.

#### Turnover

Turnover is defined as the employee’s exit from the company. It is treated as a dichotomous variable, where 1 means the employee left the company within the observed timeframe, and 0 means he or she did not leave.

#### Matching Score

Before calculating the matching score, we needed to clean and process the text data of interviewers’ narrative comments as well as the dimensions from each radar chart. Because the interviewers’ narrative comments were written in Chinese, and the Chinese words are not delimited, word segmentation was a necessary step (Peng et al., 2017). First, we used a Chinese stopword list to remove symbols and common words that did not provide any insights (e.g., “the,” “that,” “here,” etc.) from the content. Second, Python programming language and a Chinese Natural Language Processing package HanLP (Han He, 2014) was used to segment sentences in the comments and job-analysis dimensions. Sentences in comments were broken down into meaningful words, which were then to match with the words from the job-analysis dimensions. The matching score was calculated according to the formula:

$$\frac{\sum_{i\in D}S_{i}*Occur_{i}}{\sum_{i\in D}S_{i}}$$

where D referred to the dimensions from the radar chart, $S_{i}$ referred to the weight of ith dimension, and $Occur_{i}$ referred to the number of occurrence of ith dimension in the comments. Since the radar chart for each job has a different number of dimensions with different weights, we normalized the matching score by dividing the sum of all the dimension weights.

#### Breadth Coverage

The breadth of dimensions from the radar chart interviewers covered during the interview is calculated by adding how many dimensions appeared in the comments. More specifically, the algorithm goes through all the phrases in the comments one by one. Whenever a new dimension that has not been seen in the previous phrases appears, the breadth coverage number adds one. Then the number is divided by the total number of dimensions for normalization since each radar chart has a different number of dimensions. Therefore, the number represents the total number of unique dimensions the interviewer has been able to assess during the interview. Although the breadth number is calculated using the comments written after the interview, it is still a relatively accurate measure. The company requires interviewers to write comments after each interview, which will become a company record for both the interviewer and the interviewee if the candidate is hired. Since this is part of interviewers’ job, they are responsible to write it to their best knowledge. With different writing styles, the length of the comments may vary across interviewers. However, these comments should reflect the ideas and dimensions interviewers assessed during the interview, at least what impressed the interviewer the most which led to his/her decision of whether hire or pass.

#### Controls

A candidate’s performance after he or she is selected into the company may be influenced by many factors. Therefore, several control variables were included in the analyses for better estimates of our hypotheses. We used a dichotomous variable as an indicator of the employee’s gender, where 1 indicated male and 0 indicated female because male employees may be favored more than female employees, especially in a male-dominated work environment such as in our case. Employee education level is added as a control variable because different education levels will have different impacts on the employee’s ability to process complex information, therefore affect his or her job performance (Wally and Baum, 1994). Workplace age stereotypes are getting more prevalent in affecting performance evaluation, promotion, and retention of employees (Posthuma and Campion, 2009). Thus, we included employee age as a control variable.

Moreover, the average team member rating was included as a control variable, where we aggregated all team members’ performance ratings and divided them by the total number of members. Team sizes were controlled because they affect how well team members know each other for performance grading. Different job clans may have different performance rating criteria, such that employees in some clans may more easily take credits for outstanding outputs, while others may not be so obvious. Therefore, we controlled the job position as well. A dummy variable of the year of hire was also added to reflect the changing environment of the labor market as well as the business the company was running in.

## Results

Descriptive statistics, the means, SD, and correlations among all variables are presented in Table 1. To test our hypotheses, we used employees’ age, education level, gender, the size of their team, the year of hire, and the team’s average performance score as control variables. The matching score between interviewer narrative comments and dimensions on the radar chart was used as the independent variable to test its effects on performance, promotion, and turnover. We used a three-way interaction between matching score, breadth coverage, and the Clan of the candidate to test the moderating effect of breadth coverage on matching score in different job Clans (Mitchell, 2012). Tables 2–4 provide the results of multivariate tests of our hypotheses for the dependent variables of performance, promotion, and turnover, respectively. Specifically, model 1 only took the control variable as an independent variable, while model 2 tested the direct effect of matching score on performance and promotion using Ordinary Least Squares (OLS) regression analysis, and turnover using Logit modeling. In addition to model 2, model 3 added a three-way interaction to test hypotheses 2 and 3. The coefficients are shown in Tables 2–4, with the asterisk indicating the significance level.

**Table 1:** Means, SD, and correlations continued.

Variables	Mean	SD	Min	Max	(1)	(2)	(3)	(4)	(5)	(6)	(7)	(8)	(9)	(10)	(11)
(1) Performance	3.163	0.748	1	5	1.00										
(2) Promotion	0.164	0.736	0	7	0.09	1.00									
(3) Turnover	0.00327	0.0571	0	1	−0.11	−0.00	1.00								
(4) Breadth coverage	9.042	3.094	1	20	0.04	0.03	−0.00	1.00							
(5) Matching score	2.638	1.685	1	21.30	0.04	0.06	−0.01	0.29	1.00						
(6) Age	31.16	4.184	20.50	58.50	0.06	0.09	0.00	−0.02	0.13	1.00					
(7) Education	3.345	0.600	1	5	0.03	0.01	0.01	0.06	0.06	0.12	1.00				
(8) Male	0.754	0.431	0	1	0.01	0.03	0.00	0.07	0.08	0.07	−0.01	1.00			
(9) Hire year	24.14	1.510	11	26	−0.01	−0.06	−0.01	0.05	−0.03	0.03	0.05	0.06	1.00		
(10) Team size	10.98	7.178	1	62	−0.01	−0.02	0.01	−0.01	−0.01	−0.09	−0.04	0.12	0.03	1.00	
(11) Team mean performance	3.313	0.276	1	5	0.32	0.03	−0.06	0.04	0.01	0.02	0.05	−0.00	0.02	−0.00	1.00

*N* = 7,650.

**Table 2:** Statistics analysis results for dependent variable performance.

Variable	Performance		
	Model 1	Model 2	Model 3
Age	0.009^***^	0.009^***^	0.009^***^
Education	0.007	0.011	0.011
Male	0.011	0.016	0.013
Team size	−0.001	−0.001	−0.001
Hire year	−0.011^**^	−0.011^**^	−0.012^**^
Team mean performance	0.862^***^	0.862^***^	0.863^***^
Matching score		0.012^**^	
Job Clan controls		Yes	
Design Clan^*^Matching score^*^Breadth			0.139
Marketing Clan^*^Matching score^*^Breadth			0.020
Product/Project Clan^*^Matching score^*^Breadth			−0.078
Specialists Clan^*^Matching score^*^Breadth			−0.071
Technical Clan^*^Matching score^*^Breadth			0.011
_cons	0.271	0.199	0.201
N	7650	7650	7650
Adjusted $R^{2}$	0.104	0.107	0.107
△$R^{2}$		7.44^***^	2.10

* *p* < 0.1;

** *p* < 0.05;

*** *p* < 0.01.

**Table 3:** Statistics analysis results for dependent variable promotion.

Variable	Promotion		
	Model 1	Model 2	Model 3
Age	0.015^***^	0.017^***^	0.018^***^
Education	0.004	−0.001	−0.002
Gender	0.058^***^	0.019	0.019
Team size	−0.002	−0.003^**^	−0.003^**^
Hire year	−0.033^***^	−0.036^***^	−0.035^***^
Team mean performance	0.065	0.064	0.060
Matching score		0.016^**^	
Job Clan controls		Yes	
Design Clan*Matching score*Breadth			0.010
Marketing Clan*Matching score*Breadth			0.053
Product/Project Clan*Matching score*Breadth			−0.171^***^
Specialists Clan^*^Matching score^*^Breadth			−0.099^*^
Technical Clan^*^Matching score^*^Breadth			−0.106
_cons	0.240	0.103	0.186
N	7650	7650	7650
Adjusted $R^{2}$	0.013	0.022	0.029

* *p* < 0.1;

** *p* < 0.05;

*** *p* < 0.01.

**Table 4:** Statistics analysis results for dependent variable turnover.

Variable	Turnover		
	Model 1	Model 2	Model 3
Age	0.000	−0.004	−0.017
Education	0.487	0.444	0.378
Gender	0.115	−0.034	−0.096
Team size	0.034^**^	0.032^*^	0.038^**^
Hire year	−0.115	−0.126	−0.137
Team mean performance	−2.492^***^	−2.563^***^	−2.604^***^
Matching score		−0.233^***^	
Job Clan controls		Yes	
Design Clan^*^Matching score^*^Breadth			0.000
Marketing Clan^*^Matching score^*^Breadth			−1.628
Product/Project Clan^*^Matching score^*^Breadth			−2.917
Specialists Clan^*^Matching score^*^Breadth			−9.913^***^
Technical Clan^*^Matching score^*^Breadth			−3.225^***^
_cons	3.262	4.032	−1.476
N	7650	7650	7650

* *p* < 0.1;

** *p* < 0.05;

*** *p* < 0.01.

For candidates’ performance, model 1 in Table 2 showed that age and average team performance were positively related to performance rating, while the year of hire was negatively related to performance with significance. When the matching score was added to the model, the performance rating coefficient was positive and statistically significant (*β* = 0.012, *p* < 0.05) with increased Adjusted $R^{2}$ value, supporting Hypothesis 1a. In addition, an F-test was carried out in model 2 with the result of a significant F-change, meaning that matching score significantly improved the prediction of the variables. One of the problems when dealing with a large sample size is that *p*-values approaching zero, giving statistical significance but impractical effects. Zhang et al. (2010) calculated the marginal effect in their study to show practical significance. Following their method, we calculated the marginal effect of matching scores on performance. When the matching score increases by one SD (SD = 1.685), performance would increase by 2 percent.

For the promotion dependent variable, model 1 in Table 3 showed that age and gender were positively related to the number of promotions, while the year of hire was negatively related to promotion with significance. In model 2, where the matching score was added, the coefficient was positive and statistically significant (*β* = 0.016, *p* < 0.05) with increased Adjusted $R^{2}$ value, supporting Hypothesis 1b.

Model 1 in Table 4 showed that team size was positively related, and average team performance was negatively related to turnover with significance. Supporting Hypothesis 1c, model 2 with the matching score added indicates turnover indicator coefficient was negative and statistically significant (*β* = −0.233, *p* < 0.01), suggesting that the higher the matching score, the less likely the candidate would leave the company. Since we used Logit modeling for the turnover dependent variable, no Adjusted $R^{2}$ value was reported.

To test Hypotheses 2 and 3, we used a three-way interaction between matching score, breadth coverage, and Clan. High breadth coverage indicates that the interviewer covered more dimensions from the radar chart in the questions asked during the interview, while low breadth coverage indicates fewer dimensions covered. The analysis was performed using Stata, following the examples and instructions in Mitchell (2012) for continuous by continuous by categorical interactions. Results are shown in model 3s in Tables 2–4 for the three dependent variables analyzed respectively. For candidates’ performance ratings, our results indicated that none of the coefficients for the three-way interaction terms was significant for all five Clans, thus failed to support our Hypotheses 2a and 3a.

**Figure 2:**
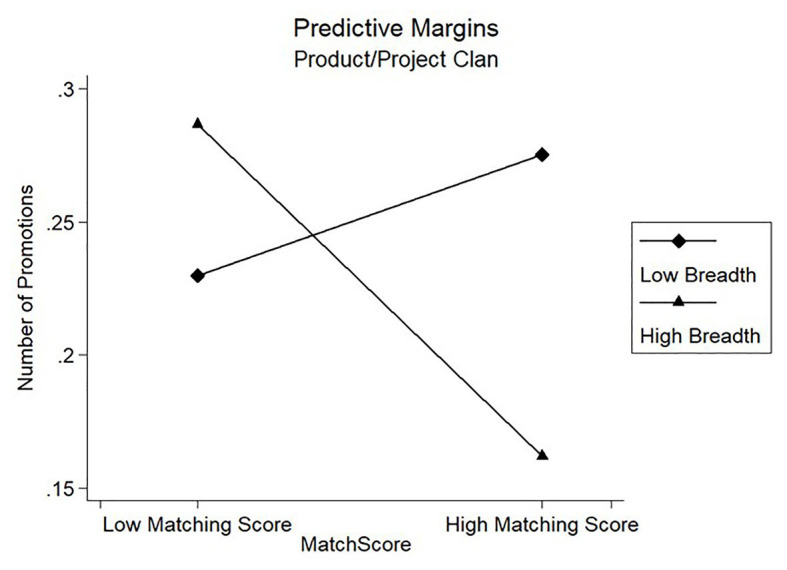
Moderating effect of breadth coverage on promotion for Product/Project Clan.

**Figure 3:**
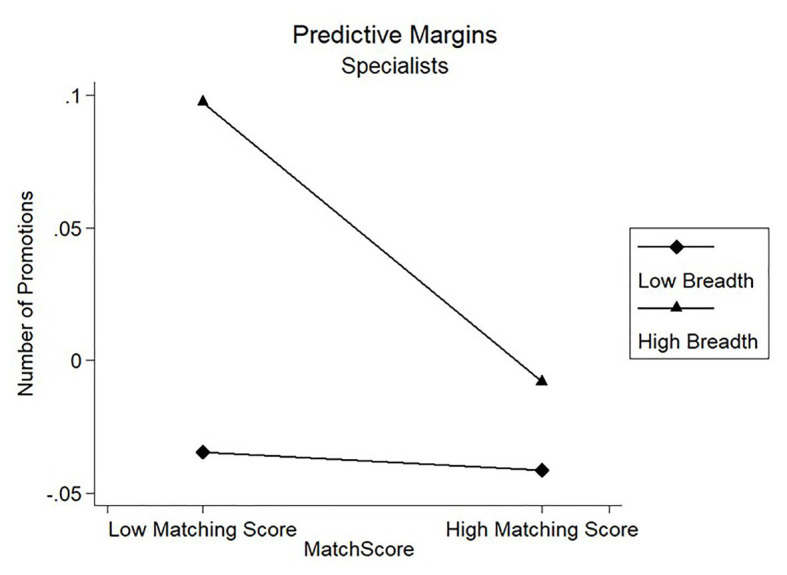
Moderating effect of breadth coverage on promotion for Specialists Clan.

Results of model 3 in Table 3 showed that the coefficients of the interaction term on candidates’ number of promotions were negatively significant for the Product/Project Clan (*β* = −0.171, *p* < 0.01) and the Specialists Clan (*β* = −0.099, *p* < 0.1). Figures 2, 3 showed the marginal prediction of the breadth coverage for different levels of matching score on the number of promotions for the Product/Project Clan and the Specialists Clan, respectively. As seen in Figure 2, the fewer dimensions covered with higher matching score gave a better prediction on candidates’ number of promotions. However, Figure 3 shows that low breadth resulted in negative marginal prediction for Specialists Clan, suggesting that low breadth coverage caused fewer promotions. These results suggested that the fewer job-analysis dimensions interviewers covered during the interview, the better the matching score predicted candidates’ number of promotions, partially supporting Hypothesis 2b but failing to support Hypothesis 3b.

**Figure 4:**
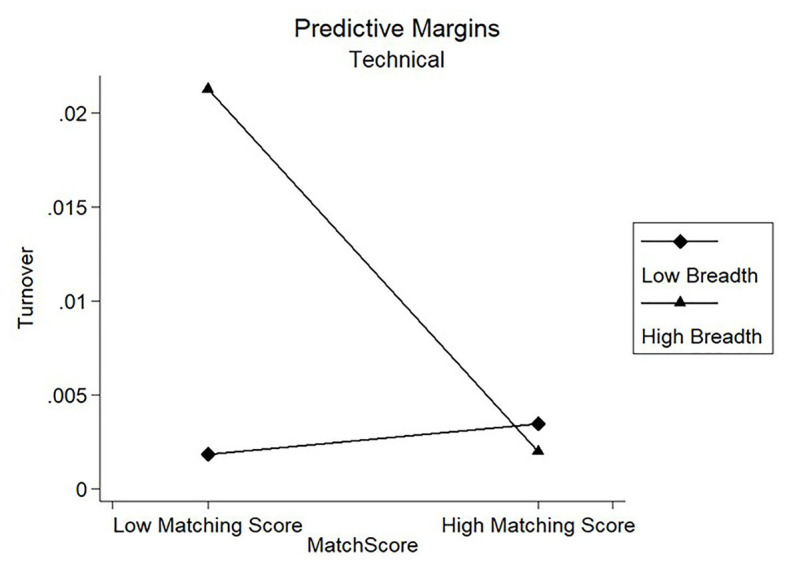
Moderating effect of breadth coverage on turnover for Technical Clan.

**Figure 5:**
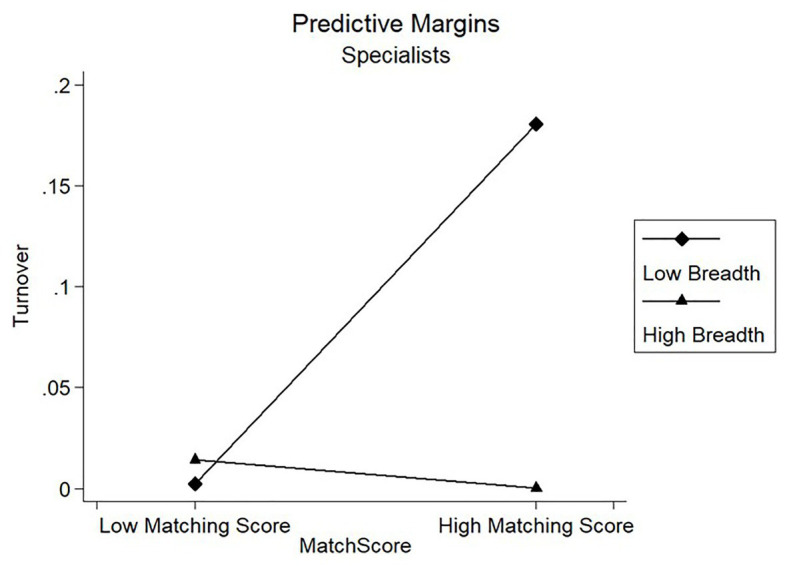
Moderating effect of breadth coverage on turnover for Specialists Clan.

Results of model 3 in Table 4 showed that the coefficients of the interaction term on candidates’ turnover were negatively significant for the Specialists Clan (*β* = −9.913, *p* < 0.01) and negatively significant for the Technical Clan (*β* = −3.225, *p* < 0.01). Figures 4, 5 showed the marginal prediction of the breadth coverage for different levels of matching score on turnover for the Specialists Clan and the Technical Clan, respectively. As observed from Figure 4, low breadth coverage was comparatively better than high breadth coverage for Technical Clan, such that candidates interviewed with fewer dimensions were less likely to leave the company. However, Figure 5 shows that low breadth coverage predicted higher turnover than high breadth coverage as the matching score increased for Specialists Clan. These results partially supported our Hypotheses 2c but failed to support our Hypotheses 3c.

## Discussion

The results of our study show that the match between interviewer’s narrative comments and capability dimensions on the radar chart had a significant effect on interview validity in the candidate’s job performance, promotion, and turnover. The candidate’s job performance after being selected is the common measure of interview validity (Huffcutt and Arthur, 1994; Kleinmann et al., 2011). However, most of the results were drawn using mock interviews and experimental data (e.g., Ingold et al., 2015; Huffcutt et al., 2017). Our study further confirmed the current literature empirically by using data from a real work environment. While promotion is often considered a result of higher performance ratings (Lyness and Heilman, 2006), our results indicate a direct relationship between interview qualities and the number of promotions. Despite the theoretical implication that DA fit influences employee turnover (Kristof-Brown et al., 2005), whether interview qualities have a direct effect on interviewee turnover was hard to analyze because of the lack of data from real scenarios. Our results showed and confirmed this theoretical implication empirically, that is, that DA fit decreased turnover rate. Together with the effect on interviewee performance and promotion, we contributed to the PJ fit literature, specifically DA fit, the finding that interview is an effective method for assessing DA fit, and thus a valid predictor for interviewees’ job performance, promotion, and turnover.

Moreover, we found that the breadth of job-analysis dimensions coverage moderates the effect of the match between interviewers’ narrative comments and job-analysis dimensions on interview validity for job categories that require deeper and narrower knowledge. Even though radar charts from job analysis is a valid tool for interview question generation, it is often treated dichotomously as to whether it is used for interview questions or not, showing a failure to appreciate the extensive amount of information it may contain. Our findings suggested an explicit usage of radar charts from job analysis in selection interviews, therefore filled the gap in knowledge of how job analysis could be better utilized to assist interviewers and extended the literature on job analysis in human resources management.

One of the reasons that our findings did not support the moderating effect of the number of job-analysis dimensions on candidates’ job performance in Hypotheses 2a and 3a was that the number of dimensions might not reflect how good the choice of dimensions by the interviewer was. Speer et al. (2019) find in their research that interviewers’ social intelligence and general mental ability are important factors that help interviewers choose more suitable interview questions and rate prospective employees accurately. Including interviewers’ personality and intelligence data may help fill this gap, and show how the different aspects of a radar chart should be chosen to predict candidates’ performance more accurately.

The moderating effects of breadth coverage on candidates’ number of promotions were significant on Specialists Clan and Product/Project Clan in Hypothesis 2b, but not on Technical Clan. On the other hand, the moderating effects were significant on Specialists Clan and Technical Clan but not on Product/Project Clan in Hypothesis 2c. This was probably because some of the jobs in the Technical and Product/Project Clan required not very deep knowledge, but broad interaction skills like jobs in Design and Marketing Clans. For example, there were jobs in the Product/Project Clan that dealt with game operations and overseas collaboration management. In the Technical Clan, jobs such as services management and operation planning were less technical but required more personal skills to coordinate with other departments. Therefore, it would be better to further test Hypotheses 2b and 2c with more specific job positions to see more clearly how the breadth coverage would moderate the results.

The failure to support Hypotheses 3b and 3c, where we argued that the higher the number of dimensions covered in the interview, the better its predictability, indicated that for jobs that required broader knowledge, the use of job analysis in interviews might require more attention. Though the job analysis is a valid component for a selection interview, as shown in Hypotheses 1a, 1b, and 1c, the number of dimensions covered might not be good enough to reveal how interviewers could utilize it in interviews for jobs in Design and Marketing Clans. Future research could focus on the content of the dimensions and how they relate to performance evaluation or PJ fit of the candidate, rather than just DA fit.

Apart from the theoretical contributions mentioned above, our results extend the literature on selection interviews by using a new type of data – interviewers’ narrative comments – as well as employees’ actual performance data within the company, to illustrate the direct effect of asking job-related questions on interview validity. Moreover, the use of text analysis on interview narrative comments is a method that has not been used in the selection interview literature, even though it is gaining more attention in other areas of human resources management research such as performance appraisal (e.g., Brutus, 2010; Speer, 2018; Speer et al., 2018), applicants’ justice perceptions (Walker et al., 2015), and training of interviewers (Shantz and Latham, 2012). Our study extends selection interview literature by introducing a new method proven in another field of study. In addition, using interviewer comments and radar charts from job analysis is a more objective method for interview candidates’ fit assessment, since it does not require employees to give ratings of the fit they perceive, which are likely to be inaccurate because of the lack of knowledge or understanding of the job requirements. This method showed a more promising fit assessment method and confirmed the extant research findings.

Even though Huffcutt and Arthur (1994) proposed a framework for different levels of interview structure in terms of the level of standardization and restriction, many studies still treat interview structure dichotomously without considering the different effects imposed by different structure levels. On the other hand, although structured interviews are believed to outperform unstructured interviews in many aspects by academic researchers, they are not well adopted by professionals and practitioners (Van der Zee et al., 2002). One of the most prominent reasons that interviewers are reluctant to use structured interviews is the lack of autonomy (Nolan and Highhouse, 2014). The contribution of our study here is practical. Our results indicate that interviewers could use job-analysis as a structure during the interview without using pre-determined questions, as long as they can form a conclusion about the candidate’s capabilities related to the job-analysis. This relaxed interview structure similar to level 2 of question standardization in framework of Huffcutt and Arthur (1994) was a valid predictor for interviewees’ performance. Using this relaxed structure is itself a contribution because most of the structured interview research has only examined whether structures are utilized in interviews but not to what degree they are utilized (Chapman and Zweig, 2005). Our study further confirms the validity of this framework with large sample size and real-world data.

Practically, our results indicate that interviewers could choose a less restricted interview structure and be more autonomous in their interviews without compromising their interview validity. Moreover, the choice of interview questions in our study was decided by interviewers without prespecified rules, which made our result more relatable to other interviewers in practice. As Zhang et al. (2018) point out, the adoption of interview structure depends on how well practitioners and professionals understand the validity of this practice. In addition, the study by Van der Zee et al. (2002) shows that a subjective norm is one of the factors that motivate interviewers what interview techniques to use. Therefore, by using data and comments from actual interviewers and how they behaved during interviews, our results would improve interviewers’ understanding and appreciation of the superior quality of a structured interview so that they are more willing to adopt this method.

## Limitations and Future Research

This study has several limitations that may need further exploration. Despite the innovative usage of interviewers’ narrative comments and real-world data generated from daily human resources activities in a real business entity, our data were drawn from one single organization, which might not generalize well to other organizations. Since different organizations may have different rules for conducting interviews and selection standards differ across industries, it would be of theoretical and practical value to compare interviewer narrative comments from other organizations and industries to see the boundary and effectiveness of how well interviewers’ narrative comments can predict interviewees’ job performance.

One of the main factors in our study is the interviewer. Whether the personality, training, and other backgrounds of the interviewer affect how he/she writes the comments and conducts the interview may have a direct effect on how well the matching score predicts the job outcome as well as the moderator effects of the breadth coverage. This issue needs to be addressed in future research from the interviewers’ perspective to provide a full scope of the study.

The requirement of the existence of high-quality radar charts from job analysis for every position might be a challenge for other organizations, especially smaller enterprises that do not have enough budget for job-analysis development. This requirement may limit the generalizability of our findings. However, what attributes interviewers are most interested in may be revealed through a thorough analysis of their narrative comments. Future research may explore the content of interviewer narrative comments using more sophisticated natural language processing techniques such as machine learning (e.g., Campion et al., 2016) to discompose the comments into different job-related attributes and explore their predictability on interviewees’ job performance. This approach will further contribute to the job-analysis literature by showing a new method of job-analysis development for selection interviews. Through the content analysis of interviewer narrative comments, organizations can figure out what attribute has the best predictive validity and construct radar charts accordingly, which also requires less time and budget.

While, we showed that a relaxed interview structure similar to level 2 in Huffcutt and Arthur (1994) was a valid predictor for interviewee job performance, we did not compare the validities of different levels of structure, given the promising usage of narrative comments data. Different interview structure levels may lead to different comments interviewers give after each interview. It is worth analyzing these differences in comment content and styles and how they impact the predictive validity of interviews. Future research could extend this study by controlling the interview structure levels to compare how they influence the comments interviewers write and which level gives the highest predictive validity.

Despite these limitations, the present study contributes to the selection interview literature by taking an important step toward addressing calls for research that investigates the validity of structured interviews, using new techniques and data that meet the shifts in selection literature that are affected by tremendous changes in business (Ployhart et al., 2017). Our sample contained employees from various positions with large sample size, as well as data from real selection interviews and employees’ job performance ratings in an actual organization setting, thus increasing our confidence in both theoretical and practical contributions. Also, our use of text analysis techniques allowed us to investigate how the match between interviewers’ narrative comments and radar chart dimensions predicts candidates’ performance and career in the organization. To our knowledge, this is the first empirical examination of how the breadth of job-analysis dimensions asked during an interview influences the interview validity. Thus, the findings of our study offer important insights into how interviewers should conduct their interviews using radar charts from job analysis for better interview validity.

## Data Availability Statement

The data will not be available to public since they are proprietary data that belong to the company.

## Ethics Statement

The studies involving human participants were reviewed and approved by South China University of Technology. Written informed consent for participation was not required for this study in accordance with the national legislation and the institutional requirements.

## Author Contributions

YC carried out the study, analyzed the results, and finished the manuscript. JJ contributed to the design and conception of the study. SL and HM helped with data acquisition and provided valuable feedback on the drafts. HZ helped with data processing and modeling. All authors contributed to the article and approved the submitted version.

### Conflict of Interest

The authors declare that the research was conducted in the absence of any commercial or financial relationships that could be construed as a potential conflict of interest.

## References

[ref2] Barrick M. R. Parks-Leduc L. (2019). Selection for fit. Annu. Rev. Organ. Psych. Organ. Behav. 6, 171–193. 10.1146/annurev-orgpsych-012218-015028

[ref3] Barrick M. R. Zimmerman R. D. (2009). Hiring for retention and performance. Hum. Resour. Manag. 48, 183–206. 10.1002/hrm.20275

[ref4] Boh W. F. Evaristo R. Ouderkirk A. (2014). Balancing breadth and depth of expertise for innovation: a 3M story. Res. Policy 43, 349–366. 10.1016/j.respol.2013.10.009

[ref5] Boon C. Biron M. (2016). Temporal issues in person–organization fit, person–job fit and turnover: the role of leader–member exchange. Hum. Relat. 69, 2177–2200. 10.1177/0018726716636945, PMID: 27904171 PMC5117123

[ref6] Brutus S. (2010). Words versus numbers: a theoretical exploration of giving and receiving narrative comments in performance appraisal. Hum. Resour. Manag. Rev. 20, 144–157. 10.1016/j.hrmr.2009.06.003

[ref7] Cable D. M. Judge T. A. (1997). Interviewers' perceptions of person–organization fit and organizational selection decisions. J. Appl. Psychol. 82, 546–561. 10.1037/0021-9010.82.4.546, PMID: 9378683

[ref8] Campion M. C. Campion M. A. Campion E. D. Reider M. H. (2016). Initial investigation into computer scoring of candidate essays for personnel selection. J. Appl. Psychol. 101, 958–975. 10.1037/apl0000108, PMID: 27077525

[ref10] Campion M. A. Palmer D. K. Campion J. E. (1997). A review of structure in the selection interview. Pers. Psychol. 50, 655–702. 10.1111/j.1744-6570.1997.tb00709.x

[ref11] Chapman D. S. Zweig D. I. (2005). Developing a nomological network for interview structure: antecedents and consequences of the structured selection interview. Pers. Psychol. 58, 673–702. 10.1111/j.1744-6570.2005.00516.x

[ref12] Conway J. M. Jako R. A. Goodman D. F. (1995). A meta-analysis of interrater and internal consistency reliability of selection interviews. J. Appl. Psychol. 80, 565–579. 10.1037/0021-9010.80.5.565

[ref13] Edwards J. R. (1991). “Person-job fit: a conceptual integration, literature review, and methodological critique” in International review of industrial and organizational psychology. Vol. 6 eds. Cooper C. L. Robertson I. T. (Oxford, England: John Wiley & Sons), 283–357.

[ref14] Elam C. L. Johnson M. Wiese H. J. Studts J. L. Rosenbaum M. (1994). Admission interview reports: a content analysis of interviewer comments. Acad. Med. 69(Suppl. 10), S63–S65. 10.1097/00001888-199410000-00044, PMID: 7916831

[ref15] Fleming L. (2001). Recombinant uncertainty in technological search. Manag. Sci. 47, 117–132. 10.1287/mnsc.47.1.117.10671

[ref17] Greve H. R. Taylor A. (2000). Innovations as catalysts for organizational change: shifts in organizational cognition and search. Adm. Sci. Q. 45, 54–80. 10.2307/2666979

[ref19] Han He (2014). HanLP: Han language processing. Available at: https://github.com/hankcs/pyhanlp (Accessed September 13, 2019).

[ref20] Hu J. Wayne S. J. Bauer T. N. Erdogan B. Liden R. C. (2016). Self and senior executive perceptions of fit and performance: a time-lagged examination of newly-hired executives. Hum. Relat. 69, 1259–1286. 10.1177/0018726715609108

[ref21] Huffcutt A. I. Arthur W. (1994). Hunter and hunter (1984) revisited: interview validity for entry-level jobs. J. Appl. Psychol. 79, 184–190. 10.1037/0021-9010.79.2.184

[ref22] Huffcutt A. I. Conway J. M. Roth P. L. Stone N. J. (2001). Identification and meta-analytic assessment of psychological constructs measured in employment interviews. J. Appl. Psychol. 86, 897–913. 10.1037/0021-9010.86.5.897, PMID: 11596806

[ref23] Huffcutt A. I. Culbertson S. S. Goebl A. P. Toidze I. (2017). The influence of cognitive ability on interviewee performance in traditional versus relaxed behavior description interview formats. Eur. Manag. J. 35, 383–387. 10.1016/j.emj.2016.07.007

[ref24] Ingold P. V. Kleinmann M. König C. J. Melchers K. G. Van Iddekinge C. H. (2015). Why do situational interviews predict job performance? The role of interviewees’ ability to identify criteria. J. Bus. Psychol. 30, 387–398. 10.1007/s10869-014-9368-3

[ref25] Kleinmann M. Ingold P. V. Lievens F. Jansen A. Melchers K. G. König C. J. (2011). A different look at why selection procedures work: the role of candidates’ ability to identify criteria. Organ. Psychol. Rev. 1, 128–146. 10.1177/2041386610387000

[ref26] Kobayashi V. B. Mol S. T. Berkers H. A. Kismihok G. Den Hartog D. N. (2018). Text mining in organizational research. Organ. Res. Methods 21, 733–765. 10.1177/1094428117722619, PMID: 29881248 PMC5975701

[ref27] Kristof-Brown A. L. Zimmerman R. D. Johnson E. C. (2005). Consequences of individuals'fit at work: a meta-analysis of person–job, person–organization, person–group, and person–supervisor fit. Pers. Psychol. 58, 281–342. 10.1111/j.1744-6570.2005.00672.x

[ref28] Levashina J. Hartwell C. J. Morgeson F. P. Campion M. A. (2014). The structured employment interview: narrative and quantitative review of the research literature. Pers. Psychol. 67, 241–293. 10.1111/peps.12052

[ref29] Lyness K. S. Heilman M. E. (2006). When fit is fundamental: performance evaluations and promotions of upper-level female and male managers. J. Appl. Psychol. 91, 777–785. 10.1037/0021-9010.91.4.777, PMID: 16834505

[ref30] Macan T. (2009). The employment interview: a review of current studies and directions for future research. Hum. Resour. Manag. Rev. 19, 203–218. 10.1016/j.hrmr.2009.03.006

[ref31] Markoulli M. P. Lee C. I. S. G. Byington E. Felps W. A. (2017). Mapping human resource management: reviewing the field and charting future directions. Hum. Resour. Manag. Rev. 27, 367–396. 10.1016/j.hrmr.2016.10.001

[ref32] Mitchell M. N. (2012). Interpreting and visualizing regression models using Stata. College Station, Tex: Stata Press.

[ref33] Morgeson F. P. Spitzmuller M. Garza A. S. Campion M. A. (2016). Pay attention! The liabilities of respondent experience and carelessness when making job analysis judgments. J. Manag. 42, 1904–1933. 10.1177/0149206314522298

[ref34] Muchinsky P. M. Monahan C. J. (1987). What is person-environment congruence? Supplementary versus complementary models of fit. J. Vocat. Behav. 31, 268–277. 10.1016/0001-8791(87)90043-1

[ref35] Nolan K. P. Highhouse S. (2014). Need for autonomy and resistance to standardized employee selection practices. Hum. Perform. 27, 328–346. 10.1080/08959285.2014.929691

[ref36] Peng H. Cambria E. Hussain A. (2017). A review of sentiment analysis research in Chinese language. Cogn. Comput. 9, 423–435. 10.1007/s12559-017-9470-8

[ref37] Platanou K. Mäkelä K. Beletskiy A. Colicev A. (2018). Using online data and network-based text analysis in HRM research. J. Organ. Effect. People Perform. 5, 81–97. 10.1108/JOEPP-01-2017-0007

[ref38] Ployhart R. E. Schmitt N. Tippins N. T. (2017). Solving the supreme problem: 100 years of selection and recruitment at the journal of applied psychology. J. Appl. Psychol. 102, 291–304. 10.1037/apl0000081, PMID: 28125261

[ref39] Posthuma R. A. Campion M. A. (2009). Age stereotypes in the workplace: common stereotypes, moderators, and future research directions. J. Manag. 35, 158–188. 10.1177/0149206308318617

[ref40] Posthuma R. A. Morgeson F. P. Campion M. A. (2002). Beyond employment interview validity: a comprehensive narrative review of recent research and trends over time. Pers. Psychol. 55, 1–81. 10.1111/j.1744-6570.2002.tb00103.x

[ref42] Sanchez J. I. Levine E. L. (2001). “The analysis of work in the 20th and 21st centuries” in International handbook of work and organizational psychology. Vol. 1 eds. Anderson N. Ones D. S. Sinangil H. K. Viswesvaran C. (Thousand Oaks, CA: Sage), 71–89.

[ref43] Sanchez J. I. Levine E. L. (2012). The rise and fall of job analysis and the future of work analysis. Annu. Rev. Psychol. 63, 397–425. 10.1146/annurev-psych-120710-100401, PMID: 21961945

[ref44] Schilling M. A. Vidal P. Ployhart R. E. Marangoni A. (2003). Learning by doing something else: variation, relatedness, and the learning curve. Manag. Sci. 49, 39–56. 10.1287/mnsc.49.1.39.12750

[ref45] Shantz A. Latham G. P. (2012). Transfer of training: written self-guidance to increase self-efficacy and interviewing performance of job seekers. Hum. Resour. Manag. 51, 733–746. 10.1002/hrm.21497

[ref46] Simonton D. K. (2003). Scientific creativity as constrained stochastic behavior: the integration of product, person, and process perspectives. Psychol. Bull. 129, 475–494. 10.1037/0033-2909.129.4.475, PMID: 12848217

[ref47] Speer A. B. (2018). Quantifying with words: an investigation of the validity of narrative-derived performance scores. Pers. Psychol. 71, 299–333. 10.1111/peps.12263

[ref48] Speer A. B. Christiansen N. D. Laginess A. J. (2019). Social intelligence and interview accuracy: individual differences in the ability to construct interviews and rate accurately. Int. J. Sel. Assess. 27, 104–128. 10.1111/ijsa.12237

[ref49] Speer A. B. Schwendeman M. G. Reich C. C. Tenbrink A. P. Siver S. R. (2018). Investigating the construct validity of performance comments: creation of the great eight narrative dictionary. J. Bus. Psychol. 34, 747–767. 10.1007/s10869-018-9599-9

[ref50] Thorsteinson T. J. (2018). A meta-analysis of interview length on reliability and validity. J. Occup. Organ. Psychol. 91, 1–32. 10.1111/joop.12186

[ref51] Van der Zee K. I. Bakker A. B. Bakker P. (2002). Why are structured interviews so rarely used in personnel selection? J. Appl. Psychol. 87, 176–184. 10.1037/0021-9010.87.1.176, PMID: 11916211

[ref52] Walker H. J. Helmuth C. A. Feild H. S. Bauer T. N. (2015). Watch what you say: job applicants’ justice perceptions from initial organizational correspondence. Hum. Resour. Manag. 54, 999–1011. 10.1002/hrm.21655

[ref53] Wally S. Baum J. R. (1994). Personal and structural determinants of the pace of strategic decision making. Acad. Manag. J. 37, 932–956. 10.2307/256605

[ref54] Wiesner W. H. Cronshaw S. F. (1988). A meta-analytic investigation of the impact of interview format and degree of structure on the validity of the employment interview. J. Occup. Psychol. 61, 275–290. 10.1111/j.2044-8325.1988.tb00467.x

[ref55] Zhang D. C. Highhouse S. Brooks M. E. Zhang Y. Y. (2018). Communicating the validity of structured job interviews with graphical visual aids. Int. J. Sel. Assess. 26, 93–108. 10.1111/ijsa.12220

[ref56] Zhang Y. Li H. Li Y. Zhou L. A. (2010). FDI spillovers in an emerging market: the role of foreign firms' country origin diversity and domestic firms' absorptive capacity. Strateg. Manag. J. 31, 969–989. 10.1002/smj.856

